# Early-life exposure to three size-fractionated ultrafine and fine atmospheric particulates in Beijing exacerbates asthma development in mature mice

**DOI:** 10.1186/s12989-018-0249-1

**Published:** 2018-03-14

**Authors:** Mei Mei, Haojun Song, Lina Chen, Bin Hu, Ru Bai, Diandou Xu, Ying Liu, Yuliang Zhao, Chunying Chen

**Affiliations:** 1 CAS Key Laboratory for Biomedical Effects of Nanomaterials and Nanosafety & CAS Center for Excellence in Nanoscience & Beijing Key Laboratory of Ambient Particles Health Effects and Prevention Techniques, National Center for Nanoscience and Technology of China and University of Chinese Academy of Sciences, Beijing, 100190 China; 2 Division of Nuclear Technology and Applications, Institute of High Energy Physics, Chinese Academy of Sciences, Beijing, 100190 China

**Keywords:** Early-life exposure, Particulate matter, Adulthood, Allergic asthma

## Abstract

**Background:**

Epidemiological studies have suggested that elevated levels of air pollution contribute to an increased incidence or severity of asthma. Although late-onset adult asthma seems to be more attributable to environmental risk factors, limited data is available on the impact of early-life exposure to size-fractionated ambient particulate matter (PM) on asthma in adults. We aimed to determine the effect on the development and exacerbation of asthma in the adult after the mice were exposed as juveniles to three size-fractionated ambient particulates collected from Beijing.

**Methods:**

The three size-fractionated ambient particulates were collected from urban Beijing in winter, heavily affected by traffic and coal-fired emissions. The typical morphological and major chemical components of the PM were characterized first. Oxidative stress and expression of DNA methyltransferases (DNMTs) were then examined in vitro and in the lungs of mouse pups 48 h after exposure to PM by oropharyngeal aspiration. When the exposed and control juvenile mice matured to adulthood, an antigen-induced asthma model was established and relevant bio-indices were assessed.

**Results:**

PM with different granularities can induce oxidative stress; in particular, F1, with the smallest size (< 0.49 μm), decreased the mRNA expression of DNMTs in vitro and in vivo the most significantly. In an asthma model of adult mice, previous exposure as juveniles to size-fractionated PM caused increased peribronchiolar inflammation, increased airway mucus secretion, and increased production of Th2 cytokines and chemokines. In general, F1 and F2 (aerodynamic diameter < 0.95 μm) particulates affected murine adult asthma development more seriously than F3 (0.95–1.5 μm). Moreover, F1 led to airway inflammation in the form of both increased neutrophils and eosinophils in BALF. The activation of the TGF-β1/Smad2 and Smad3/Stat3 signaling pathways leading to airway fibrosis was more profoundly induced by F1.

**Conclusion:**

This study demonstrated that exposure to ambient PM in juvenile mice enhanced adult asthma development, as shown by increased Th2 responses, which might be associated with the persistent effects resulting from the oxidative stress and decreased gene expression of DNMTs induced by PM exposure. The observed differences between the effects of three size-fractionated particulates were attributed to particle sizes and chemical constituents, including heavy metals and also PAHs, since the amounts of PAH associated with more severe toxicity were enriched equivalently in the F1 and F2 fractions. Relative to the often mentioned PM2.5, PM with an aerodynamic diameter smaller than 0.95 μm had a more aggravating effect on asthma development.

**Electronic supplementary material:**

The online version of this article (10.1186/s12989-018-0249-1) contains supplementary material, which is available to authorized users.

## Background

In the last few years, there has been an elevated and lasting level of air pollution in China, especially in Northern regions such as Beijing that are experiencing rapid economic growth. According to a recent comprehensive and systematic review of worldwide traffic emissions and health science by a special panel gathered by the Health Effects Institute (HEI) [1], asthma was exacerbated in children exposed to traffic-related air pollution, and this relationship has been confirmed more recently [2]. Asthma has become more common in both children and adults around the world in recent decades and is increasing rapidly especially in developing countries where the prevalence was previously low. Onset of asthma is greatest in childhood, so many articles discussed that exposure to PM during pregnancy or infancy increased the risk of offspring or childhood asthma [3–5]. However, asthma can also occur in adult without childhood asthma (late-onset adult asthma) or be from early-onset asthma persisting into adulthood. Although late-onset adult asthma seems to be more attributable to environmental risk factors, few studies have examined the persistent effect of exposure in juveniles on asthma development and exacerbation in adults. Moreover, the model we designed also emphasized a persistent role of PM exposure on asthma susceptibility and the feature of long-term impact on human health.

The incidence of asthma is increasing, and there has been a sharp increase over the last four decades [6], affecting about 300 million people worldwide [7, 8]. Asthma is also becoming increasingly prevalent in China, accompanying the high speed of industrialization as well as following especially serious air pollution in recent years. Asthma represents a chronic inflammatory disease of the airway primarily characterized by a Th2 immune response, resulting in airway hyperresponsiveness, eosinophilic inflammation, mucus production, and increased Th2 cytokines and chemokines, and even airway remodeling. Additionally, the potential role of a Th17 response in severe asthma is also highlighted [9].

Environmental factors, especially ambient air pollution, may contribute to the incidence and progression of asthma. As we know, ambient PM is a complex and heterogeneous mixture consisting of various chemical components and particulates with different sizes and surface areas. Particulates with smaller diameters are able to transport a large number of toxic components and penetrate more deeply into the alveoli, even to extra-pulmonary tissue. Therefore, PM from different sources and with different sizes causes differential toxicity resulting from multiple mechanisms. Importantly, exposure to PM at different ages leads to different immunological responses and has differing impacts on asthma development as reported [10].

In the present study, we examined the long-term effects of ambient PM exposure in juvenile mice on the development of asthma in adults. We observed that exposure to PM in mouse pups induced oxidative stress and changes in the mRNA expression of DNA methyltransferases (DNMTs). When these mice grew to adulthood and were challenged by OVA allergen, they exhibited increased peribronchiolar inflammation, airway mucus secretion, elevated Th2 responses and increased activation of signaling pathways involved in airway fibrosis. Thus, we hypothesized that early-life ambient PM exposure elicited a persistent immune response through oxidative stress and epigenetic changes and then promoted the development of asthma in mature mice.

## Results

### Characterization of ambient particulate matter in Beijing

**Fig. 1:**
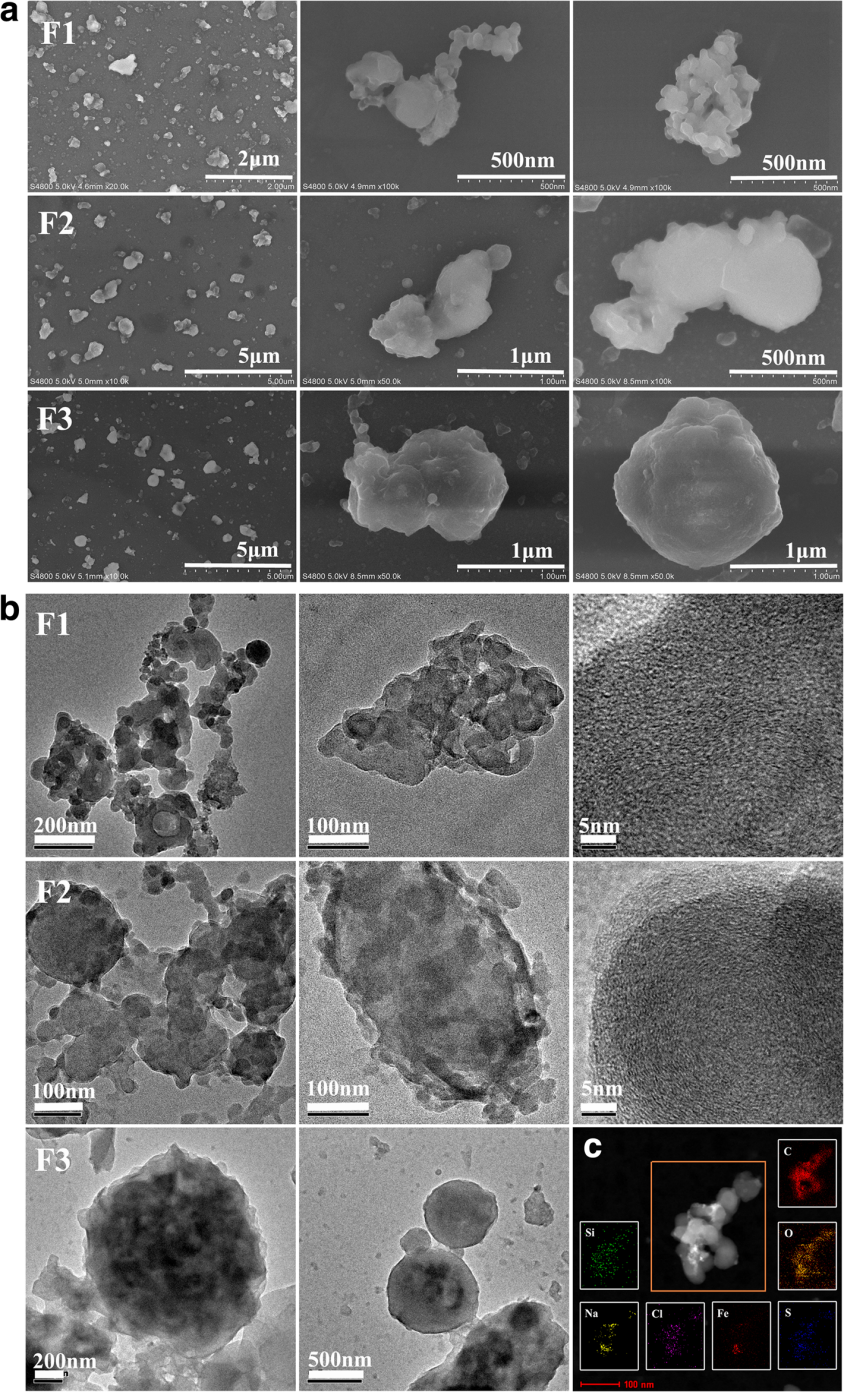
Characteristics of PM. Scanning electron micrographs (**a**) and transmission electron microscopy (**b**) were used to characterize the external morphology and internal features of the fractions of collected particulate matter. **c** Typical TEM images and relevant EDX spectra of elemental mapping results of F1

The size-fractionated atmospheric ultrafine and fine particulate matter (PM) employed and extracted in this study is referred to as F1 (< 0.49 μm), F2 (0.49–0.95 μm) and F3 (0.95–1.5 μm) according to its aerodynamic diameter. The fractions of PM contained very low levels of endotoxin that can be neglected. The particle size distribution and typical morphological features of the collected PM were detected with scanning electron micrographs (SEM) as shown in Fig. 1a. From their apparent appearance, the PM particulates extracted from filters were composed of numerous irregular and random shapes of aggregated or agglomerated particles with different sizes. The morphology of a single particle showed that it was partly an aggregate of smaller sized particles, especially F1, which had many small bead-like structures on its surface. However, particles of larger sizes, especially of F3, mostly appeared as spherical structures with uneven surfaces.

The inner microstructure of ultrafine and fine particles was investigated using transmission electron microscopy (TEM). In accordance with the SEM, TEM characterization showed that many F1 particles were irregular and composed of small bead-like structures, which presumably were grown from very tiny bead-shaped particles. Notably, a number of carbon nanostructures containing onion shell-like rings with structural defects were observed in separate F1 particles [11]. Most F2 particles appeared as a larger core structure attached to a number of smaller bead structures; similarly, some F2 particles with the onion ring-like carbon microstructure were discovered. Most F3 particles tended to have a spherical structure without the onion shell-like microstructure (Fig. 1b). Typical F1 nanoparticles consisted of the elements of carbon (C), oxygen (O), and a small amount of silicon (Si), sulfur (S), chlorine (Cl), iron (Fe) and sodium (Na) according to the elemental mapping results (Fig. 1c).

In addition to morphological features, the PM was analyzed for water-soluble trace metal species and organic PAH. A total of thirty-five elements in the PM were quantified. As shown in Table 1, the main species are S, K, Ca, Ti, P, Fe, Mg, Al and Zn. The concentrations of Ti, P, Pb, Se, Ni and Li were higher in F1; Ca, Fe, Mg, Al, Zn, Cu, Cr, As, Sr and Co had a higher enrichment in F3; and the contents of Ba, Sb, Sn and Rb in F1 and F3 were slightly higher than those in F2.

**Table 1:** The concentrations (μg/g) of trace metals in PM (mean ± SD)

	F1	F2	F3
S	49,583.9 ± 1112.2	50,692.3 ± 5236.4	57,164.0 ± 2584.2
K	11,494.5 ± 371.0	10,601.9 ± 226.6	9449.2 ± 75.1
Ca	6307.2 ± 324.3	7740.1 ± 580.5	16,752.0 ± 1037.5
Ti	5568.8 ± 384.0	2662.4 ± 223.4	3377.9 ± 91.9
P	4301.3 ± 186.9	3586.2 ± 246.6	3667.0 ± 155.4
Fe	3870.9 ± 129.7	4971.2 ± 360.3	9860.8 ± 360.4
Mg	2337.8 ± 236.1	1964.7 ± 249.2	4207.8 ± 231.2
Al	1740.8 ± 20.1	2543.4 ± 817.8	6317.7 ± 196.1
Zn	1024.9 ± 52.8	1003.0 ± 86.2	1450.6 ± 63.7
Mn	378.7 ± 20.3	463.8 ± 17.8	535.5 ± 9.4
Ba	224.3 ± 8.5	138.8 ± 53.1	276.6 ± 12.2
Pb	695.4 ± 38.7	514.9 ± 123.6	549.5 ± 9.4
Cu	395.6 ± 12.6	360.4 ± 134.3	1029.4 ± 48.0
Cr	130.7 ± 14.6	116.3 ± 50.6	194.2 ± 105.2
Sb	66.4 ± 2.2	53.7 ± 16.5	72.3 ± 3.9
Se	61.3 ± 0.6	37.3 ± 5.5	33.1 ± 3.0
As	58.1 ± 1.7	48.7 ± 17.2	99.6 ± 10.9
Sn	54.9 ± 2.5	40.6 ± 9.9	55.1 ± 2.1
Ni	53.1 ± 5.0	34.9 ± 9.8	44.8 ± 0.03
Sr	39.3 ± 1.4	44.3 ± 20.8	176.0 ± 26.1
Li	34.0 ± 2.1	25.4 ± 1.5	19.3 ± 0.8
Rb	30.2 ± 0.7	29.1 ± 11.7	37.6 ± 0.8
Mo	20.2 ± 0.2	14.7 ± 4.0	30.9 ± 1.0
Cd	14.8 ± 0.5	12.8 ± 4.4	21.2 ± 7.0
Co	13.0 ± 1.1	12.3 ± 5.9	21.6 ± 3.3
Ce	9.8 ± 0.5	4.6 ± 0.2	12.6 ± 0.4
W	9.4 ± 0.1	10.0 ± 4.5	18.3 ± 0.6
Cs	3.6 ± 0.1	3.4 ± 1.4	4.6 ± 0.1
Ag	3.0 ± 0.3	2.4 ± 0.9	4.1 ± 0.3
Pd	0.9 ± 0.5	0.3 ± 0.1	0.8 ± 0.2
Gd	0.6 ± 0.1	0.5 ± 0.1	1.2 ± 0.1
Th	0.3 ± 0.1	0.2 ± 0.1	1.1 ± 0.01
U	0.1 ± 0.04	0.2 ± 0.1	0.6 ± 0.1
Pt	0.1 ± 0.1	0	0.1 ± 0.01
Eu	0.1	0.04 ± 0.01	0.1

**Fig. 2:**
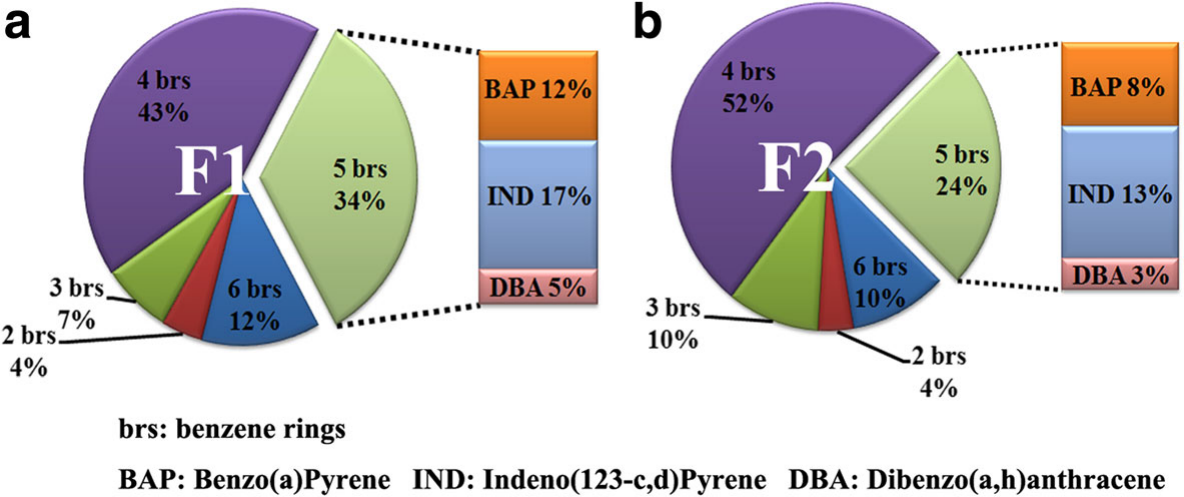
The proportion of PAH with different numbers of benzene rings in F1 (**a**) and F2 (**b**). The proportion of PAHs with two to six benzene rings was determined; the proportions of BAP, IND and DBA with five benzene rings, which may be associated with more severe toxicity, are also listed separately at the side

As shown in Table 2, the order of the total amounts of 16 PAHs in each PM fraction was as follows: F2 (1664.6 μg/g) > F1 (1187.4 μg/g) > F3 (676.7 μg/g). Specifically, the concentration of PAHs with four aromatic rings was highest in F2 (870.4 μg/g); the total amount of PAHs with five benzene rings was similar in F1 (402.5 μg/g) and F2 (405.8 μg/g) and was approximately twice as high as that in F3 (255.1 μg/g). Furthermore, the proportion of the PAH concentration that contained five benzene rings was 33.9% in F1, which was higher than the 24.4% in F2. Fig. 2 also shows the proportion of the three different components of PAH that had five benzene rings.

**Table 2:** The contents (μg/g) of 16 signature PAHs in the fractions of PM

	F1	F2	F3
Naphthalene	35.0	50.3	74.6
Acenaphthylene	1.6	2.0	1.3
Acenaphthene	2.1	1.1	0.6
Fluorene	11.3	7.6	4.4
Phenanthrene	36.6	41.0	17.8
Anthracene	2.2	2.4	0.7
Fluoranthene	45.2	115.4	42.3
Pyrene	43.1	118.7	36.6
Benzo(a)anthracene	64.6	161.6	23.8
Chrysene	96.2	192.4	51.2
Benzo(b)Fluoranthene	269.7	348.9	121.5
Benzo(k)Fluoranthene	32.6	48.9	46.8
Benzo(a)Pyrene	143.1	137.2	68.9
Indeno(123-c,d)Pyrene	205.6	216.9	92.8
Dibenzo(a,h)anthracene	53.9	51.7	19.1
Benzo(g,h,i)Perylene	144.8	168.6	74.4

Note:Determination of PAH concentrations in PM. Contents of sixteen PAHs with 2 to 6 benzene rings in fractions of PM were evaluated using GC-MS

### PM induced oxidative stress in vitro and in vivo

**Fig. 3:**
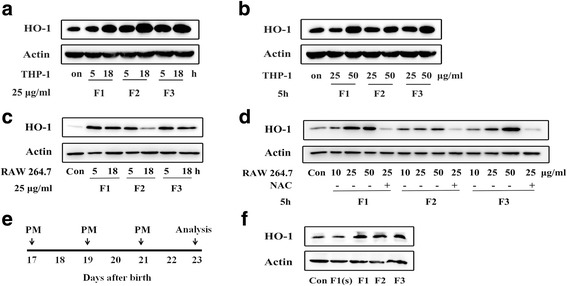
PM induced oxidative stress in vitro and in vivo. **a** and **b** The increased expression of heme oxygenase (HO-1) in THP-1 cells induced by PM at different exposure times and concentrations was determined. **c** and **d** The effects of PM on HO-1 expression in RAW 264.7 cells at different exposure times and concentrations. HO-1 expression was increased significantly following a 5 h exposure to PM (F1, F2, and F3, 25 μg/ml). PM induced HO-1 expression in a dose dependent manner, and the effect was inhibited by the antioxidant NAC. Cells were treated with PM (10, 25, or 50 μg/ml) for 5 h. RAW 264.7 cells were incubated with 20 mM NAC for 1 h before addition of 25 μg/ml PM for 5 h. **e** and **f** Schematic of PM exposure protocol. PM (F1, F2 and F3: 50 μg per dose; F1(s): 15 μg per dose) or PBS was administered by oropharyngeal aspiration to juvenile BALB/c mice on 17, 19 and 21 days after birth. HO-1 expression in lung tissues was measured by western blotting 48 h after the last exposure to PM or PBS. *n* = 3–5 mice/group

In addition to its function in heme catabolism, heme oxygenase-1 (HO-1) protein is a very sensitive biological oxidative stress marker [12, 13]. Here HO-1 expression was used to reflect the oxidative ability of PM containing heavy metals [14] and PAH [15] in vitro and in vivo. In THP-1 cells, the expression of HO-1 was increased by exposure to PM including F1, F2, and F3, in a time- and concentration-dependent manner (Fig. 3a and b). In addition, a significant increase in HO-l expression was observed in RAW 264.7 cells after exposure to PM including F1, F2, and F3 at a concentration of 25 μg/ml for 5 h. The effect resulting from incubation with F1 lasted for up to 18 h, but the expression of HO-1 after exposure to F2 for 18 h decreased compared with incubation for 5 h. While all three types of PM induced HO-1 expression in a dose-dependent manner, at 50 μg/ml F1 affected the response more than F2 and F3. Moreover, the antioxidant *N*-acetylcysteine (NAC) was effective in depressing the HO-1 expression induced by PM (Fig. 3c and d).

Because the pro-inflammatory activity as well as toxicity induced by PM was accompanied by oxidative stress responses [16], we asked whether PM induced oxidative stress in vivo. In agreement with the in vitro results, the expression of HO-1 was significantly increased in the mouse lung 48 h after PM exposure via three oropharyngeal aspiration (OA) doses (50 μg per dose) compared to the PBS control (Fig. 3e and f).

### Early-life exposure to PM decreased mRNA expression of DNMTs

**Fig. 4:**
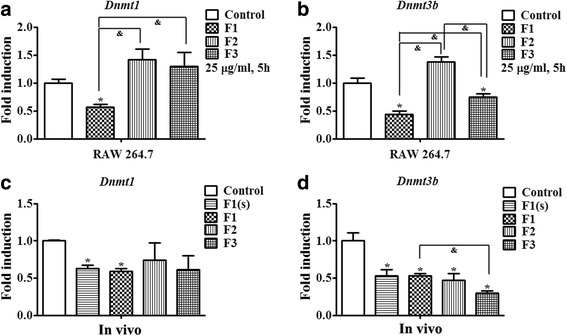
Effects of PM exposure on DNMTs in vitro and in vivo. **a** and **b** The effect of PM treatment on mRNA expression of DNMTs in vitro. RAW 264.7 cells were exposed to 25 μg/ml PM for 5 h. **c** and **d** mRNA expression of DNMTs in lung tissues of mice was assessed 48 h following exposure to PM three times on the 17th, 19th and 21st days of postnatal age. *n* = 5 mice/group. Data are expressed as mean ± SEM. *p < 0.05 vs. Con group, ^&^p < 0.05 vs. OVA/PM (F1, F2 or F3)

Because alteration of DNA methylation is closely related to asthma development [17, 18], we evaluated the impact of PM on DNMTs in vitro. Down-regulation of *Dnmt1* and *Dnmt3b* mRNA was observed in RAW 264.7 cells following exposure to 25 μg/ml F1 for 5 h. Exposure to 25 μg/ml F3 also decreased mRNA expression of *Dnmt3b* (Fig. 4a and b).

Subsequently, the lung tissues of mice exposed to PM three times by oropharyngeal aspiration (OA) were used to analyze mRNA expression of DNMTs. Consistent with the results in the cellular study, we also observed that the expression of DNA methyltransferase (*Dnmt1* and *Dnmt3b*) was significantly reduced in the lung tissues of mice after F1 exposure, and F2 and F3 exposure also statistically significantly decreased *Dnmt3b* mRNA expression as well (Fig. 4c and d).

### Early-life exposure to PM induced aggravated pulmonary inflammation and mucus production in adult mouse models of asthma

**Fig. 5:**
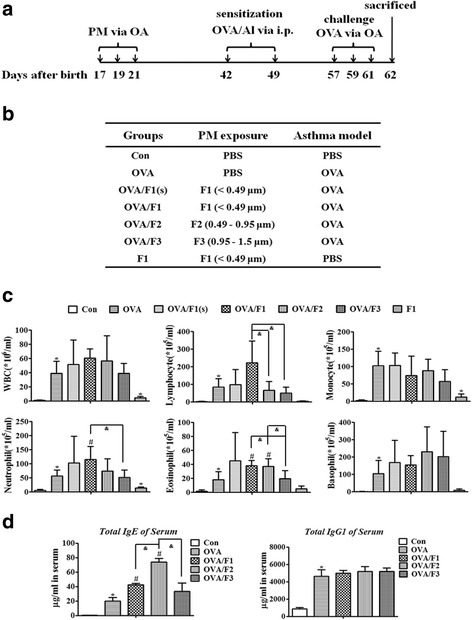
PM exposure in infant mice enhances pulmonary inflammation in adulthood after induction of allergic asthma in vivo. **a** The exposure protocol to PM and induction of asthma model. PM (F1, F2 and F3: 50 μg per time; F1(s): 15 μg per time) or PBS was administered to juvenile BALB/c mice at 17, 19 and 21 days after birth by oropharyngeal aspiration (OA). An OVA-model of asthma was established at six weeks of age. **b** The test groups are shown in the table. **c** Total WBC and the differential counts for Monocyte, Neutrophil, Lymphocyte, Basophil and Eosinophil in BALF were evaluated by differential counting 24 h following the final protocol day. **d** Serum antibody concentration measured by ELISA. The data are expressed as the mean ± SEM of 5 mice per group. *p < 0.05 vs. Con group; #p < 0.05 vs. OVA group, ^&^p < 0.05 vs. OVA/PM (F1, F2 or F3)

To determine whether exposure of mice to PM as juveniles exacerbated OVA-induced pulmonary inflammatory responses in adult mice, total lung-infiltrating and differential cell counts in bronchoalveolar lavage fluid (BALF) were quantified. As shown in Fig. 5c, the numbers of total and differential cells were all markedly increased in the OVA group compared to the PBS control. Compared to the OVA group, there were significant increases of neutrophils and eosinophils in the OVA/F1 mice, as well as an obvious increase of eosinophils in the OVA/F2 group. However, no significant upregulation of cell number in the OVA/F3 group was observed. Additionally, we found that WBC, monocytes, and neutrophils were elevated in the F1 exposure group compared to the PBS control.

Immunoglobulin production in serum was also analyzed (Fig. 5d). As expected, the levels of Total IgE and IgG1 in OVA mice were significantly higher than in PBS control mice. Total IgE concentration was statistically increased in both the OVA/F1 and OVA/F2 groups compared to OVA mice, with levels 2.1- and 3.7-fold higher, respectively. The total IgG1 concentration was increased approximately equally in the PM/OVA group and the OVA group, without statistical differences.

**Fig. 6:**
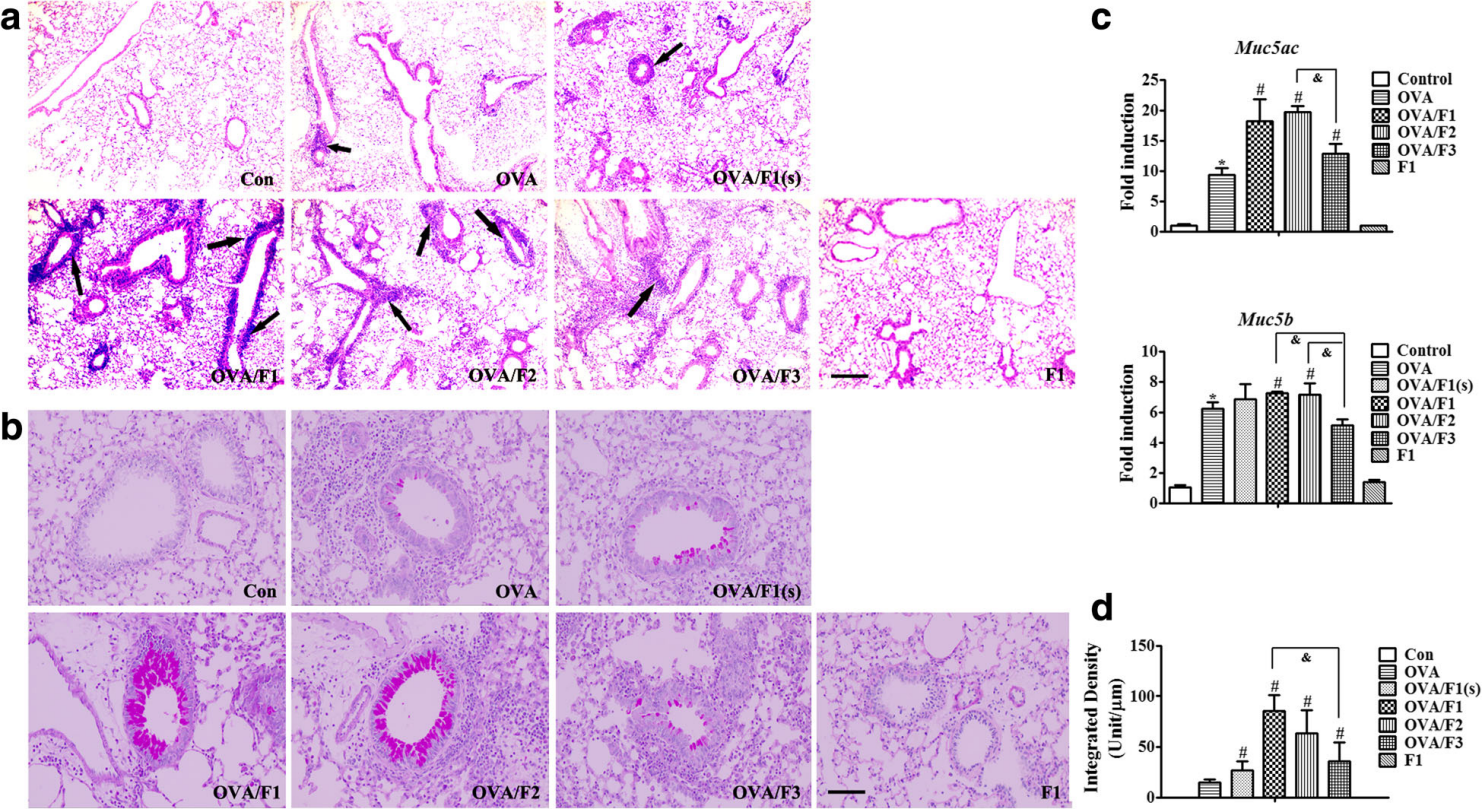
Exposure of infant mice to PM enhances pulmonary inflammation and airway mucus secretion in an asthma model. **a** Morphologic features of mouse lung inflammation stained with H&E. Arrows depict peribronchiolar inflammation. Scale bar = 50 μm. **b** Representative micrographs of PAS-stained mucus in airway epithelium. Scale bar = 100 μm. **c** The mRNA expression of *Muc5ac* and *Muc5b* in mouse lung tissues. **d** The degree of PAS staining in the airway epithelium was quantified using ImagePro-Plus 7.0 (Media Cybernetics, USA). Data are plotted as means ± SEM (*n* = 5 mice/group). *p < 0.05 vs. Con group; #p < 0.05 vs. OVA group, ^&^p < 0.05 vs. OVA/PM (F1, F2 or F3)

To further investigate the impact of PM exposure in early life on the OVA-induced asthma model in mature mice, we studied the alteration in pulmonary and mucus production at 24 h following the final OVA challenge (Fig. 5a). Lung histopathology showed an increase of inflammation infiltration in the peribronchiolar region in the OVA group compared with the PBS control (Fig. 6a). Moreover, compared to the OVA exposure mice, severe peribronchial and even perivascular inflammatory infiltration with an apparent increase was observed in both the OVA/F1 and the OVA/F2 groups. PAS staining demonstrated widespread mucus secretion in the pulmonary bronchiolar region in the OVA/PM group, and a significant increase in mucus production in the bronchioles of OVA/F1 and OVA/F2 mice was observed compared to the OVA mice (Fig. 6b). The toxicities caused by F1 were all dose-dependent; however, with respect to the PBS control, almost no change of inflammation or mucus secretion was found in the mice exposed to F1 only, without OVA.

In addition to PAS staining, mucus-associated mRNA expression in whole lung tissue was evaluated. In accordance with the result from mucus production, the expression of the mucus-related genes *Muc5ac* and *Muc5b* were obviously elevated in both the OVA/F1 and OVA/F2 groups relative to the OVA group, and OVA/F3 exposure also led to a statistical increase in *Muc5ac* expression compared to OVA mice (Fig. 6c).

### Early-life exposure to PM altered Th2 cytokine and chemokine responses in adults after induction of asthma

It has been suggested that T helper type-2 (Th2) cells and proinflammatory cytokines and chemokines play an important role in the induction and development of the inflammatory cascade in allergic asthma. Ten cytokines and chemokines (IL-4, IL-5, IL-13, IL-6, IL-10, KC, MCP-1, RANTES, IL-12p70 and IFN-γ) in BALF were measured. Relative to the OVA group, the concentrations of Th2 cytokines, including IL-4,IL-5, IL-13 as well as IL-10 [19], and the proinflammatory cytokine IL-6 were increased significantly in the BALF of OVA/F1 mice; elevated levels of IL-5 and IL-13 as well as IL-10 in the BALF of the OVA/F2 group and a higher concentration of IL-4 as well as IL-6 in the BALF of the OVA/F3 group were also observed (*p* < 0.05) (Table 3). Other BALF levels in OVA/F1 mice (Ccl chemokine MCP-1), in OVA/F2 mice (IL-4, IL-6, KC, and MCP-1), and in OVA/F3 mice (IL-5, IL-13, IL-10, and MCP-1) tended to be elevated, but without statistical differences. The levels of RANTES, IL-12p70 and IFN-γ were below the limit of sensitivity of the assay in most groups. To supplement the above data, the concentrations of cytokines and chemokines in OVA/F1 with two different doses of F1 and the F1-only group were also compared with the OVA and PBS groups in Table 3.

**Table 3:** Cytokines and chemokines in BALF (pg/ml)

	Con	OVA	OVA/F1(s)	OVA/F1	OVA/F2	OVA/F3	F1
IL-4	0.17 ± 0.06	50.93 ± 22.26^**^	149.46 ± 124.91	231.97 ± 154.35^**#**^	56.36 ± 23.12	199.42 ± 110.28^**#**^	0.60 ± 0.31^*^
IL-5	N.D.	136.66 ± 57.97	170.42 ± 134.08	253.26 ± 85.01^**#**^	244.68 ± 24.24^**#**^	138.16 ± 94.55	N.D.
IL-13	N.D.	43.05 ± 24.24	46.75 ± 42.45	79.15 ± 22.87^**#**^	71.97 ± 7.28^**#**^	59.11 ± 41.72	N.D.
IL-6	N.D.	8.86 ± 4.11	44.72 ± 37.13^**#**^	62.62 ± 50.37^**#**^	10.93 ± 3.08	53.21 ± 36.97^**#**^	N.D.
IL-10	5.09 ± 3.77	33.70 ± 16.79^*^	49.59 ± 47.22	62.09 ± 21.52^**#**^	64.86 ± 20.00^**#**^	64.41 ± 34.99	6.98 ± 5.11
KC	30.81 ± 3.20	148.15 ± 25.71^**^	110.13 ± 45.35	151.83 ± 42.91^**^	168.54 ± 106.24	113.79 ± 8.59	31.31 ± 7.76
MCP-1	N.D.	10.65 ± 3.90	15.69 ± 11.55	15.25 ± 6.13	13.12 ± 10.10	18.71 ± 11.54	N.D.

Cytokines and chemokines in mouse BALF were investigated using a Millipore mouse cytokine/chemokine magnetic bead panel. Results are expressed as mean ± SEM. n = 5–6 mice/group. ^*^p < 0.05 and ^**^P < 0.01: OVA or F1 vs. Con; ^#^p < 0.05: OVA/ F (refers to F1(s), F1, F2 or F3) vs. OVA; N.D.: non-detected

**Fig. 7:**
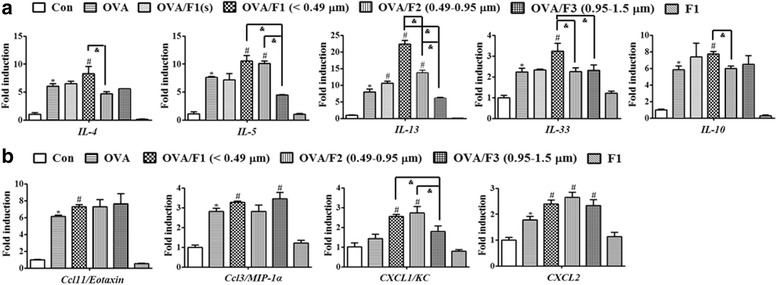
The mRNA expression levels of Th2 cytokines and chemokines in mouse lungs were measured by real-time PCR. **a** The regulation of Th2 type cytokines and *IL-33* was observed in an asthma model with mice previously exposed to PM as infants. **b** mRNA expression of eosinophil-attracting chemokine *Ccl11*, and Th2 chemokines was more strongly triggered in mouse lungs following PM exposure as infants. Data are expressed as means ± SEM (n = 5 mice/group). *p < 0.05 vs. Con group; #p < 0.05 vs. OVA group, ^&^p < 0.05 vs. OVA/PM (F1, F2 or F3)

Beside changes in the secretion levels of related proteins, we observed a series of increases of genes involved in Th2 cytokines and chemokines following exposure to PM. At the transcription level, compared with OVA mice, there was significant up-regulation of mRNA of the Th2 type cytokines *IL-4*, *IL-5* and *IL-13* (Fig. 7a) in lung tissues of OVA/F1 mice at 24 h after the last OVA challenge (schedule shown in Fig. 5a). Similarly, mRNA expression of Th2 cytokines including *IL-5* and *IL-13* in OVA/F2 mice was increased significantly relative to the OVA group. In addition, mRNA of the cytokines *IL-33* and *IL-10* was expressed at notably higher levels in OVA/F1 mice than in OVA mice. Furthermore, mRNA expression of the eosinophil-attracting chemokine *Ccl11* (*Eotaxin*) was more triggered in OVA/F1 mice than in OVA mice (Fig. 7b), and the expression levels of the chemokine *Ccl3* gene (*MIP-1α* in the mouse) in OVA/F1 and OVA/F3 mice were also significantly elevated compared with OVA mice. The transcriptional levels of chemokine (C-X-C motif) ligand 1 (*CXCL1/KC*) and *CXCL2* in the lung tissues of the OVA/F1 group were higher than those of the OVA group. Consistently, OVA/F2-induced mRNA expression of *CXCL1/KC* and *CXCL2* was markedly increased. The related mRNA expression in OVA/F1 with a lower dose of F1 and the F1-only group are also shown in Fig. 7.

### F1 exposure increased the activation of the TGF-β1/Smad2 and Smad3/Stat3 signaling pathways leading to an airway fibrosis response in adults after OVA sensitization

**Fig. 8:**
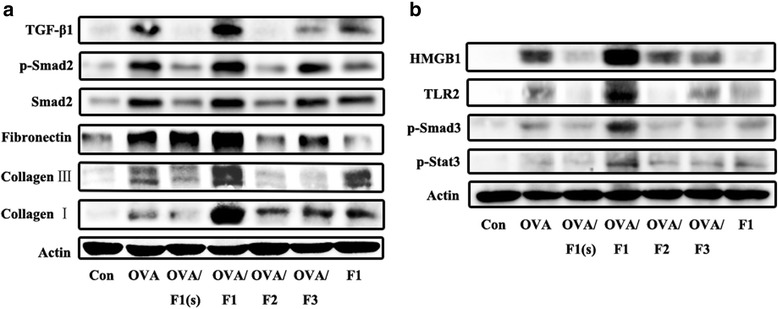
F1 exposure enhanced the OVA-induced activation of the TGF-β1/Smad2 (**a**) and Smad3/Stat3 (**b**) pathways involved in airway fibrosis. The expression of the relevant proteins was detected 24 h after the last OVA challenge according to the schedule shown in Fig. 5. n = 5 mice/group

If with a long-term recurrent asthma, a variety of cytokines may also stimulate the airway epithelial cells to secrete multiple growth factors acting synergistically on subcutaneous fibroblasts and smooth muscle cells, which induce airway epithelial fibrosis, basement membrane thickening and airway remodeling. However, asthma inflammation disassociates from fibrosis, so we next determined whether PM had a role in inducing airway fibrosis. Although fibrotic reactions were not observed in lungs stained with Masson’s Trichrome, F1 exposure-enhanced activation was seen for the TGF-β1/Smad2 [20] and Smad3/Stat3 [21] pathways that result in fibrosis (Fig. 8). In addition, expression was increased for fibronectin, collagen I and collagen III after F1 exposure.

PM can induce expression of both inflammatory and fibrogenic mediators, the two pathways were most activated by F1 fraction (Fig. 8) may not be explained comprehensively using inflammatory mechanisms of asthma analyzed above and need to be further studied.

## Discussion

**Fig. 9:**
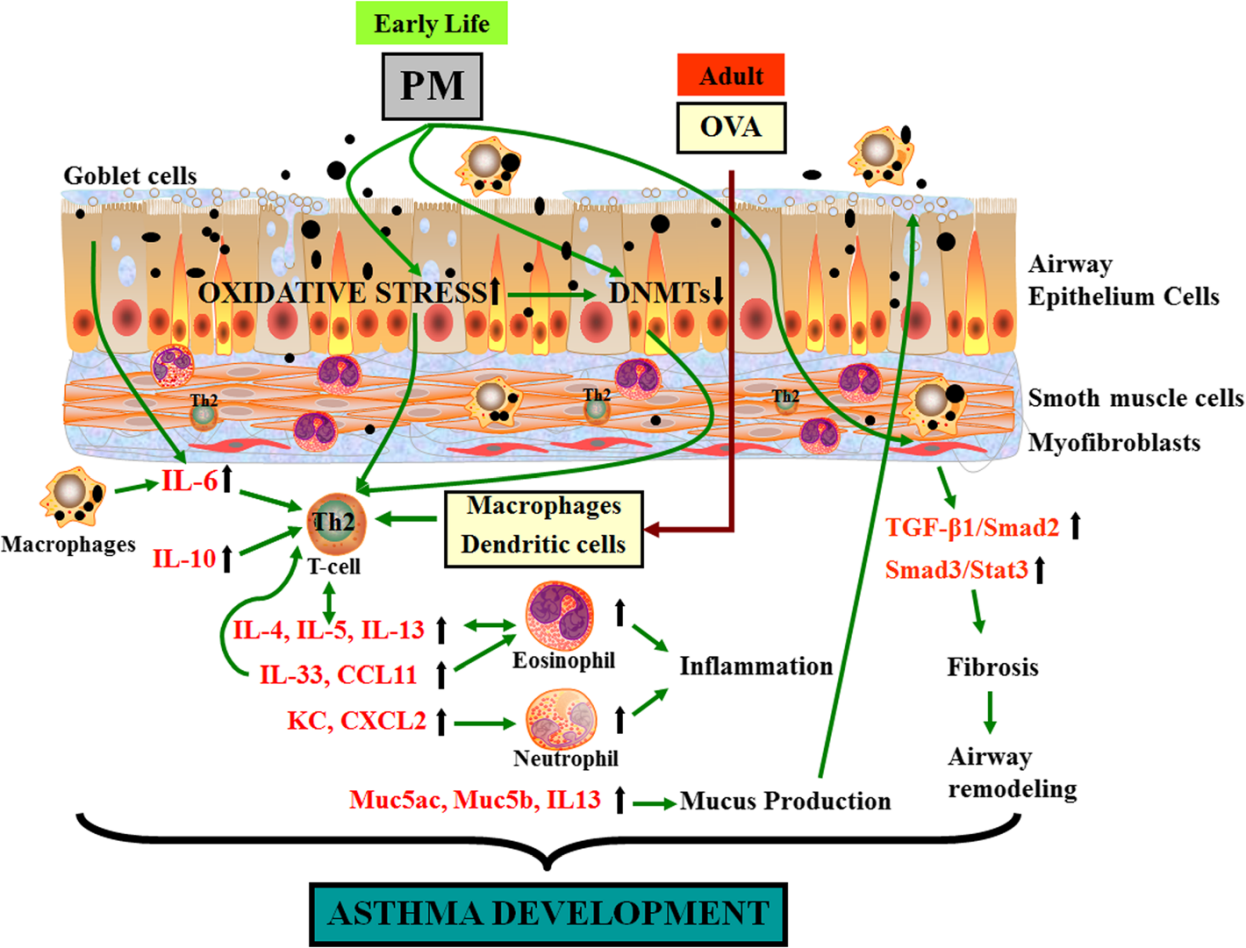
Early-life exposure to PM in Beijing exacerbates asthma development in adult mice

This study explores the effects of childhood exposure to PM found in Beijing on adult asthma development. We collected and characterized the ultrafine and fine particle matter in Beijing, then demonstrated that PM induced oxidative stress and decreased DNA methyltransferase in vitro and in vivo. Importantly, the adverse pulmonary effects of PM in mouse pups lasted into adulthood and enhanced OVA-induced asthma development in mature mice, including increased severity of lung eosinophilic inflammation and mucus secretion, together with elevated mRNA and protein levels of Th2 type cytokines and chemokines. Moreover, F1 also induced enhanced activation of the signaling pathway involved in airway fibrosis (Fig. 9). In previous research, the effects on asthma development or aggravation were mainly investigated using diesel exhaust particles [22, 23] or combustion-derived PM [24, 25] on adult or pregnant mice [26]. There is little data for size-fractionated atmospheric ultrafine and fine PM; moreover, these reports [27, 28] focus only on a model of exposure to adults. Herein we chose to expose juvenile mice to ambient PM with three different aerodynamic diameters and observed asthma susceptibility and development in them as adults.

Asthma is generally characterized by an imbalance between Th1 and Th2 immune responses and typically favors a Th2-biased response [29, 30]. Consistent with these findings, PM exposure in juvenile mice exacerbated the OVA-induced asthma response in adult mice, as evidenced by increased neutrophil and eosinophil inflammation and airway mucus secretion in lung tissues, as well as elevated levels of Th2 cytokines and chemokines in mRNA (*IL-4, IL-5, IL-13, IL-33, Ccl11/Eotaxin, Ccl3/MIP-1α, CXCL1/KC* and *CXCL2*) or protein (IL-4, IL-5, IL-13, IL-6, IL-10 and KC). Because F1 fraction contains ultrafine particles, we assume that the F1 fraction has greater ability to induce toxicity. Then in addition to comparison among PM of three particle sizes, we incorporated two different concentrations of F1 in combination with OVA into our studies to show the relationship between dose and effect, and used an F1-only group as a particle control. In summary, as the mice matured, we established an asthma model using OVA allergen and tested relevant indicators.

Our research demonstrated that F1 exposure in juveniles amplified both neutrophilic and eosinophilic inflammation compared with the OVA group, but F2 exposure in juveniles only increased eosinophilic inflammation. As reported, IL-33 is an innate cytokine from the IL-1 family, which is expressed at a higher level in asthmatic patients [31]. Due to its ability to activate Th2 and dendritic cells, IL-33 plays an important role in initiating allergic inflammation and asthma by producing Th2 cytokines including IL-5 and IL-13 [32, 33]. According to our determination, the mRNA expression level of *IL-33* in the adult lung, once exposed to F1, was significantly higher than for the OVA group. Consistently, we observed that F1 and F2 exposure augmented OVA-induced production of IL-5 and IL-13 in BALF; it also increased mRNA expression of these Th2 cytokines in lung tissues. In addition, increased mRNA expression of *Ccl11* induced by PM was noticed in our study. Experimental investigations have demonstrated that owing to the presence of CC-chemokine ligand 11 (Ccl11), an eotaxin, IL-33 may lead to eosinophil recruitment [32]. Morphometric analysis revealed that F1 and F2 exposure significantly amplified mucus production, which was compatible with increased expression of the mucus-associated genes *Muc5ac* and *IL-13* in the lungs of F1 and F2 exposed mice. We also found that the gene expression of neutrophil chemoattractants, including *CXCL1* and *CXCL2*, which are inducible by IL-17A and regarded as Th17 signature genes [34], was increased in the lungs of adult mice that had once been exposed to PM as juveniles. Although allergic asthma has always been considered to be a Th2 disease with increased eosinophils and Th2 cytokines such as IL-4, IL-5 and IL-13, severe asthma related to clinical resistance may result from a mixed Th2/Th17 response [35]. Compared to mice exposed only to OVA, we found that the combination with F1 exposure as juveniles increased the mRNA expression of *IL-10* in the adult lung, which can result in the production of more Th2 cells to prolong the immune response [34]. A slightly increased expression of the *GATA3* gene in the lungs was also observed 48 h after PM exposure (Additional file 1: Figure S1B). *GATA3*, a transcription factor of Th2, selectively stimulates the growth of Th2 cells and suppresses Th1 differentiation [36]. Because of binding to the promoters of IL-5 and IL-13, deleting the *GATA3* gene may block IL-5 and IL-13 production [37]. Additionally, decreased expression of *MyD88* mRNA resulting from PM exposure was observed (Additional file 1: Figure S1B). As demonstrated before, the airway allergic response induced by OVA in MyD88^- /-^ NOD mice was drastically exacerbated [38]. Collectively, there was a positive correlation between the presence of eosinophils, Th2 cytokines, mucus secretion and airway inflammation, suggesting multiple effects stimulated by airborne PM in the pathophysiology of asthma.

Due to the complexity and variability of the components in ambient PM, the mechanism by which PM exposure skews Th2 cytokine production and contributes to antigen-induced adult asthma development remains not fully understood. Some investigations have suggested that oxidative stress [39] and epigenetic change [40] mediate the toxicity and adverse effects of PM. There is accumulating evidence that oxidative stress plays a role in the pathogenesis of asthma [41] and is central to the process of augmenting allergen-induced lung inflammation [42]; oxidative stress has also been shown to mediate the adjuvant effect of PM. Epigenetic modification, including changes in DNA methlylation, histone modifications, and miRNA, has been reported to induce Th2 polarization along with associated cytokines and chemokines involved in asthma development [43, 44]. DNMTs have been shown to combine with the signature gene loci of Th2 cytokines or chemokines through interaction with key transcriptional factors, thus controlling their expression [45].

To determine whether the effect of ambient PM, lasting from juvenile to adult, in mice can be correlated with the different oxidative potential or epigenetic remodeling, we measured HO-1 as a marker of oxidative stress and the mRNA expression of DNMTs as a sign of epigenetic change. Our results demonstrated that size-fractionated PM increased the expression of HO-1 at the cellular level, exhibitive of the oxidative potential. Consistent with the data in vitro, increased expression of HO-1 was also observed in the lung tissues of mouse pups 48 h after PM exposure. On the other hand, genes involved in inflammation and the immune response may be altered by DNMTs. As shown previously, the expression of DNMTs in the lungs of asthmatic mice was altered compared to normal mice [17]. We next determined whether PM exposure linked with changes of mRNA expression of genes encoding proteins played a role in regulating DNMTs. Specifically, DNMT3a and DNMT3b are responsible for de novo methylation, and DNMT1 is also important for maintaining the methylation pattern [46, 47]. Decreased expression of *Dnmt1* [48, 49] and *Dnmt3b* mRNA in mouse lungs was detected after PM exposure. This result was in agreement with the down-regulation of *Dnmt1* mRNA after exposure to F1 (25 μg/ml) in RAW264.7 cells. The expression of *Dnmt3b* mRNA after exposure to F1 in RAW264.7 cells was also significantly diminished. Thus, ambient PM exposure seemed to cause a primed state where these mice in the growing process became hypersensitive to subsequent allergen exposure. Some clinical and epidemiological evidence indicates the effect of epigenetic changes in childhood on long-term health outcomes such as asthma [50–52]. These studies are consistent with our findings that the expression of DNMTs [53] was decreased in juvenile mice exposed to PM, and subsequent Th2 responses were exacerbated when these mice grew up and encountered antigen, indicating that PM may lead to long-term adverse effects on health.

We speculated that an irregular surface such as “strings of beads” of PM with smaller particle sizes characterized by electron micrographs, especially F1 and F2, may be related to their elevated ability to accumulate transition metal and organic ingredients such as PAH. Transition metals in ambient PM, which usually originate from anthropogenic activities, may catalyze oxidative potential [54]. Specifically, Ni, which was enriched mostly in F1, can influence the respiratory system, then lead to inflammation and asthma. Previous research has indicated that ultrafine particles of TiO_2_ cause more severe inflammation and epithelial damage than fine TiO_2_ particles of equal mass [55]. Most potent activation of fibrosis pathway by F1 may be related to high content of Ti in F1 fraction. As reported, Pb has the ability to disrupt the prooxidant/antioxidant balance, even causing oxidative bursts [56, 57]. Investigations have shown that Pb and Se are associated with daily mortality in six US cities [58]. Apart from resulting in oxidative stress, heavy metals can also affect DNMTs [59]. For instance, the toxic effects of Ni have been associated with the ability to cause DNA methylation and histone modification [60]. Pb can inhibit DNMTs [61], and epigenetic changes following Pb exposure in early life may increase the susceptibility to disease in the long term [62]. The toxic effects of Cu, Fe and Cr may also be associated with their effect on epigenetic regulation [63, 64]. It is worth noting that the oxidative potential of metals may be related to the valence state which will need to be further verified. PAHs are complex mixtures of components having two or more aromatic rings, and there is a different structure-activity relationship for each different chemical in PAHs [65]. As reported, in PAHs with five benzene rings, benzo(a)pyrene (BaP) and dibenzo(a,h)anthracene as well as indeno(123-c,d)pyrene display more potent immunotoxicity and carcinogenicity [66]. Furthermore, BaP has been suggested to cause DNA methylation modification [67]. For our determination, although F2 had the largest total content of all 16 PAHs taken together, F1 and F2 had equivalent enrichment of the PAHs with five benzene rings, which was 2.2-fold higher than that in F3. As a percentage of total PAHs, F1 had the highest proportion with five benzene rings at 33.9%, compared with 24.4% in F2 and 26.7% in F3. These reports are in accordance with the toxicity resulting from size-fractionated PM exposure in our studies. PAHs contained in ambient PM may drive oxidative stress, which plays a significant role in leading to allergic airway disease [27, 68]. Additionally, exposure to air pollution, especially PAHs, has also been associated with DNA methylation changes [69]. PAHs can cause Th2 polarization and related cytokine and chemokine up-regulation mediated through epigenetic modification [70].

Previous research has shown that PM2.5 has a greater impact on health than coarse particles do, and now PM2.5 as a main indicator is routinely monitored in China and elsewhere. However, few studies have focused on which size of particle is most closely related to the development and exacerbation of asthma. Because F4 (1.5–3 μm) had the weakest ability to induce asthma in our preliminary test, we used PM with three smaller aerodynamic diameters, which we designated F1 (< 0.49 μm), F2 (0.49–0.95 μm) and F3 (0.95–1.5 μm). Although the particle size of F3 is very close to that of F2 and F1, the effect induced by F3 was significantly reduced. Our results indicated that PM0.95 (particulate matter with aerodynamic diameter < 0.95 μm) is more closely associated with asthma than PM2.5, consistent with the latest epidemiological study [71]. We also estimated PM mass concentration which, when inhaled, would produce a human dose equivalent to the dose mice were exposed to in this research. According to this calculation [72], concentrations of approximately 63 and 212 μg/m^3^ PM inhaled for 8 h would produce human doses equivalent to the mouse exposure doses of 15 and 50 μg, respectively. However, the most toxic PM, those with mass proportion ranges of PM0.5 and PM0.5–1, made up only 16.7–19.1% and 21.7–22.5%, respectively, of the pollution of winter days in Beijing [73]. So the adverse effect observed in the study induced by F1 or F2 should be greater than that resulting from the real ambient PM with the same mass concentration.

Collectively, our observations support the notion that oxidative stress and methylation modification induced by PM can lead to eventual changes in lung structure and can persist, thus closely linking ambient PM exposure with later asthma development in adult mice.

## Conclusions

In summary, our results indicate that ambient PM with three particle sizes of collected from winter in urban Beijing induced oxidative stress and decreased expression of DNMTs in vitro and in the lungs of juvenile mice. The observed differences between three size-fractionated PM were attributed to particle sizes and chemical constituents including heavy metals and PAHs, since the PAH amounts were quite enriched in the F1 and F2 fractions. Importantly, early-life exposure to any of the three PM fractions exacerbated asthma development in mature mice; in an adult asthma model, increased peribronchiolar inflammation, mucus production and Th2 polarization were observed in mice exposed early in life to PM, especially F1 and F2, compared to the OVA group. In addition, F1 exposure increased the activation of the signaling pathway associated with airway fibrosis. A series of the abovementioned effects might resulted from the source, particle size, specific surface area and composition of ambient PM, although the specific correspondence between these factors and their effects needs to be further determined due to the complexity and variability of ambient PM.

## Methods

### Ultrafine and fine particles

Urban particulate matter was collected, size-fractionated, for 24 h a day continuously for one month from December 1 to December 29, 2015 (with 3 to 4 days a week of a heavy haze) on the building rooftop of the National Center for Nanoscience and Technology away from the industrial area in Beijing. Briefly, the particulate matter was collected using a high volume cascade impactor TE-236 (Tisch Environmental Inc., USA) on PTFE filters (5 μm, Safelab Technology Ltd., Beijing). The TE-236 has 6 impactor stages operating at a 40 CFM inlet flow rate, with nominal cut-point diameters of 0.49, 0.95, 1.5, 3, 7.2 and 10 μm, respectively. Next, the collected particulate samples were extracted from the PTFE filters by soaking for 30 min in ultrapure water and sonicating for 30 min, followed by vortexing for 5 min as previously described [74]. Aqueous suspensions of the particulate fractions with the three smallest aerodynamic diameters, called F1 (< 0.49 μm), F2 (0.49–0.95 μm) and F3 (0.95–1.5 μm), respectively, were then used for characterization and exposure experiments.

### PM characterization

The morphology and particle size distribution of PM was examined by SEM (S-4800 N, HITACHI Inc., Japan), and the internal structure was characterized by TEM (T20, TECNAI Inc., USA); additionally, the elemental composition of F1 was detected roughly by a mapping analysis using TEM with an FEI F20 instrument. Trace heavy metals were analyzed by inductively coupled plasma-mass spectrometry (ICP-MS, X7, Thermo Electron, USA), and K, Ca, Mg, P, S, Fe, Al, Zn, Mn, and Ba by inductively coupled plasma-optical emission spectrometry (ICP-OES, Optima 8000, PerkinElmer, USA) as described previously [75]. Sixteen EPA priority PAHs were extracted and measured by GC/MS analysis (VARIAN Saturn 2000 with 3800 GC, USA) as previously described [76].

### Cell culture and exposure

RAW 264.7 cells were cultured in DMEM containing 10% FBS and penicillin/streptomycin. (San Francisco, CA). THP-1 was cultured in RPMI 1640 supplemented with 10% FBS, penicillin/streptomycin, and glutamine. Cells were plated onto six-well plates and treated with 0, 10, 25 or 50 μg/ml of PM water soluble extract. NAC with a final concentration of 20 mM was administered for 1 h before PM exposure. After incubating with PM for 5 h or 18 h, cells were harvested for detection of gene or protein expression.

### Exposure to PM and establishment of the asthma model in vivo

Female BALB/c mice (16–18 days) were purchased from Beijing Vital River Laboratory Animal Technology Co. Ltd.; all animals were housed in ventilated cages and allowed free access to food and water in a carefully controlled environment. All animal protocols were approved by the Institutional Animal Care and Use Committee at the Chinese Academy of Medical Sciences. On days 17, 19, and 21 after birth, mice were administered 50 μg PM (F1, F2 or F3) or 15 μg F1 in the OVA/F1(s) group by oropharyngeal aspiration (OA) [77, 78]. Control mice were administered the same volume of PBS. When these mice matured to adults (six weeks of age), a model of asthma was established by sensitizing and challenging with ovalbumin (OVA). Mice were divided into seven groups (*n* = 5–6 per group): Con (PBS control), OVA, OVA/F1(s), OVA/F1, OVA/F2, OVA/F3 and F1 (Fig. 5b). The OVA, OVA/F1(s), OVA/F1, OVA/F2 and OVA/F3 group mice were immunized i.p. on days 0 and 7 with a mixture of 20 μg OVA (Sigma-Aldrich, St. Louis, MO) emulsified in Imject Alum (Pierce, Rockford). Subsequently, on days 16, 18 and 20, mice were challenged with 10 μg OVA by the OA route (Fig. 5a). The Con and F1 groups were sensitized and challenged with PBS at each time point.

### BAL cellularity and cytokines

Bronchoalveolar lavage (BAL) was performed by flushing the lungs with 1 ml PBS. BAL fluid and cells were then isolated by centrifugation of the lavaged samples. Differential cell counts were performed on a fully-automatic hematology analyzer (Nihon Kohden Celltac E (MEK-7222 K)). Cytokine levels in the BALF were measured using a Milliplex mouse cytokine/chemokine 10-plex kit (Millipore Corporation, MA) according to the manufacture’s protocol on a FLEXMAP 3D System (Millipore Corporation, MA). The 10-plex consisted of IL-4, IL-5, IL-13, IL-6, IL-10, KC, MCP-1, RANTES, IL-12(p70) and IFN-γ. The sensitivity range of standards ranged from 3.2 to 10,000 pg/ml. The concentrations of cytokines detected were quantified using a standard curve, and a five-parameter logistic regression was applied to calculate the concentration of each cytokine from the unknown samples. The data shown in Table 3 excluded concentrations below the range of sensitivity for the particular analyte.

### Circulating immunoglobulin

Serum samples were analyzed for total IgE and IgG_1_ by ELISA according to the manufacturer’s directions (eBioscience, USA). ELISA plates were coated with capture antibodies overnight at 4 °C, then incubated with diluted serum (1: 400 IgE; 1: 80000 IgG_1_) and HRP-linked detection antibodies.

### Western blotting

Proteins were extracted from cells and tissues using RIPA buffer (Pierce, MA) and protein concentrations were measured using a BCA Protein Assay Kit. SDS-PAGE and Western blotting were conducted as described previously [79].

### RT-PCR analysis

Total RNA from cells and tissues was extracted and reverse-transcribed to cDNA as described previously [80]. Real-time PCR with an Eppendorf Mastercycler system (Eppendorf, Hamburg, Germany) was performed using SYBR Green PCR Master Mix (Cat. QPK-201, Toyobo, Osaka, Japan). The expression of mRNA was normalized to GAPDH. The fold induction was calculated as 2^−ΔΔCt^. The primer sequences used for PCR are shown in Additional file 1: (Table S1).

### Lung histopathology

Lung tissues were excised and fixed in 4% paraformaldehyde for 24 h. Then the fixed lungs were dehydrated, paraffin-embedded, and sectioned at 4 μm. Sections were stained with H&E and periodic acid-Schiff (PAS) to assess inflammation and mucus production, respectively. The PAS-positive area was semi-quantified using ImagePro-Plus 7.0 software by expressing the integrated density of areas as PAS-positive mucosubstances per unit length of the basement membrane [81, 82].

### Statistical analysis

Cell experiment was performed 2–3 times with three wells per group each time, and the number of animals per group was listed in the table or figure legend for each test. All results were presented as mean ± SEM. Graphs were constructed using GraphPad Prism 5 Software (GraphPad Software Inc., USA). Comparisons were performed by two-tailed Student’s t-test analysis. *P*-values of < 0.05 were considered to be statistically significant.

## Additional file

Additional file 1: **Figure S1.** mRNA expression in mouse lungs was detected 2 days after exposure to PM by OA. **Figure S2.** PM induces oxidative stress in vitro and in vivo. **Table S1.** Primers used for quantitative real time PCR analysis. **Table S2.** The parameters involved in inflammatory responses with statistical difference between three size-fractionated ultrafine and fine atmospheric particulate matter groups in OVA-induced asthma model. (PDF 2041 kb)
