# Supplementary material for: Early-life exposure to three size-fractionated ultrafine and fine atmospheric particulates in Beijing exacerbates asthma development in mature mice
Source: Part Fibre Toxicol. 2018 Mar 14;15:13. doi: 10.1186/s12989-018-0249-1 (PMC5851307; doi:10.1186/s12989-018-0249-1)
Supplement: Supplementary file 1 — Figure S1. mRNA expression in mouse lungs was detected 2 days after exposure to PM by OA. Figure S2. PM induces oxidative stress in vitro and in vivo. Table S1. Primers used for quantitative real time PCR analysis. Table S2. The parameters involved in inflammatory responses with statistical difference between three size-fractionated ultrafine and fine atmospheric particulate matter groups in OVA-induced asthma model. (PDF 2041 kb) [file 12989_2018_249_MOESM1_ESM.pdf]

Additional file

Early-life Exposure to Three Size-fractionated Ultrafine and Fine Atmospheric Particulates in Beijing Exacerbates Asthma Development in Mature Mice

Mei Mei[1], Haojun Song [2], Lina Chen [1], Bin Hu [1], Ru Bai [1], Diandou Xu [2], Ying Liu [1], Yuliang Zhao [1] and Chunying Chen [1]*

*Correspondence: chenchy@nanoctr.cn.

[1] CAS Key Laboratory for Biomedical Effects of Nanomaterials and Nanosafety & CAS Center for Excellence in Nanoscience & Beijing Key Laboratory of Ambient Particles Health Effects and Prevention Techniques, National Center for Nanoscience and Technology of China and University of Chinese Academy of Sciences, Beijing 100190, China

[2] Division of Nuclear Technology and Applications, Institute of High Energy Physics Chinese Academy of Sciences, Beijing 100190, China

A full list of author information is available at the end of the article

Additional file 1

Figure S1. mRNA expression in mouse lungs was detected 2 days after exposure to PM by OA. The gene expression of *GATA3*, a Th2 transcription factor, was elevated. In contrast, the mRNA expression of *MyD88* was decreased following PM exposure. *p < 0.05 vs. Con group (n = 4-5 mice/group).

Figure S2. PM induces oxidative stress in vitro and in vivo. (A) The increased expression of heme oxygenase (HO-1) in THP-1 cells induced by PM was determined. (B) The effects of PM on HO-1 expression in RAW 264.7 cells. Cells were treated with PM (10, 20, 50 μg/ml) for 5 h. RAW 264.7 cells were incubated with 20 mM NAC for 1 h before addition of 25 μg/ml PM for 5 h. (C) HO-1 expression in lung tissues was measured 48 h after the last exposure to PM or PBS. Induction of HO-1 expression by PM exposure was observed in vivo. n = 3-5 mice/group. The data are presented as the mean ± SEM. *p<0.05 vs. Con, #p<0.05 vs. 25 μg/ml PM without NAC, & p<0.05 vs. OVA/PM (F1, F2 or F3).

Table S1. Primers used for quantitative real time PCR analysis.

Table S2. The parameters involved in inflammatory responses with statistical difference between three size-fractionated ultrafine and fine atmospheric particulate matter groups in OVA-induced asthma model**.**

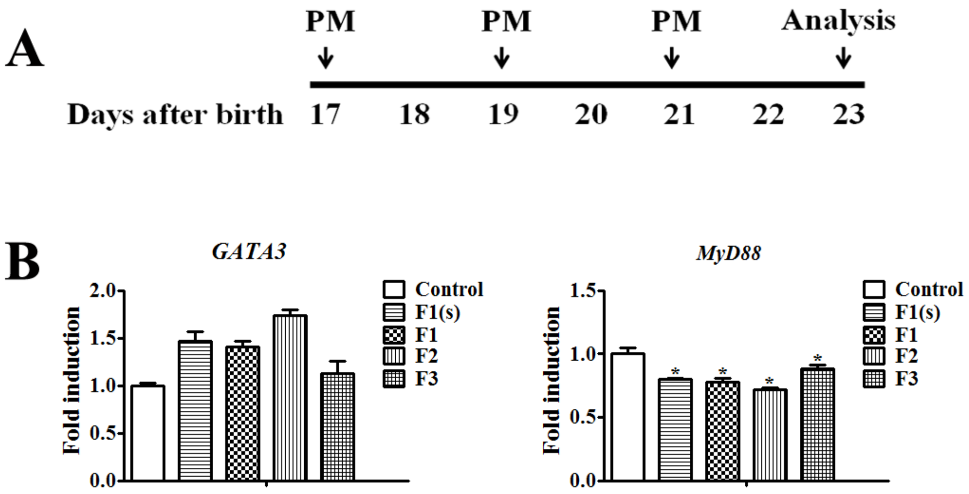

Figure S1. mRNA expression in mice lungs was detected 2 days after exposure to PM by OA. The gene expression of *GATA3*, a Th2 transcription factor, was elevated. In contrast, the mRNA expression of *MyD88* was decreased following PM exposure. *p < 0.05 vs. Con group (n = 4-5 mice/group).

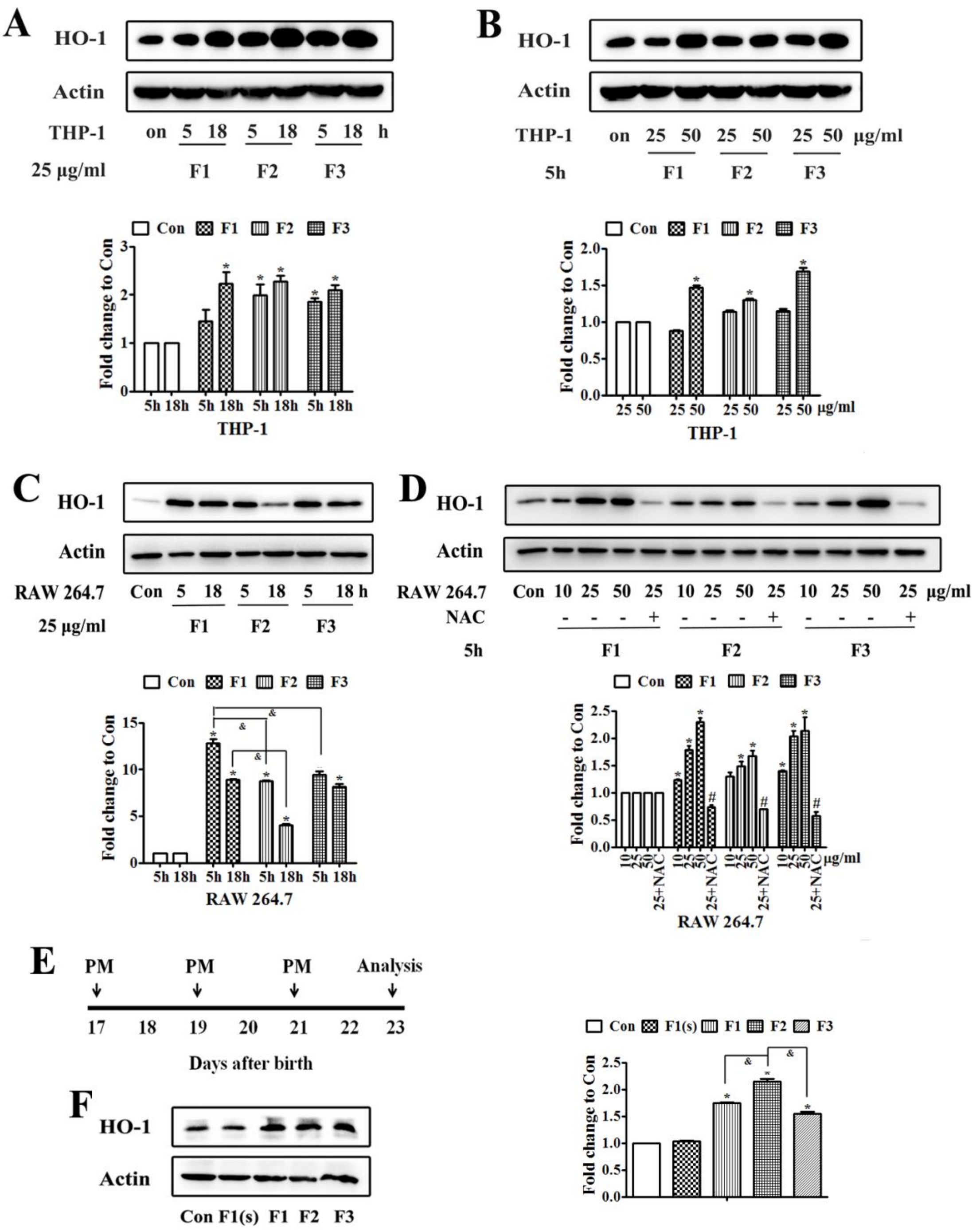

Figure S2. PM induces oxidative stress in vitro and in vivo. (A) The increased expression of heme oxygenase (HO-1) in THP-1 cells induced by PM was determined. (B) The effects of PM on HO-1 expression in RAW 264.7 cells. Cells were treated with PMs (10, 20, 50 μg/ml) for 5 h. RAW 264.7 cells were incubated with 20 mM NAC for 1 h before addition of 25 μg/ml PMs for 5 h. (C) HO-1 expression in lung tissues were measured at 48 h after the last exposure to PMs or PBS. Induction of HO-1 expression by PM exposure was observed in vivo. n=3-5 mice/group. The

data were presented as the mean ± SEM. *p < 0.05 vs. Con, #p< 0.05 vs. 25μg/ml PM without NAC, &p<0.05 vs. OVA/PM (F1, F2 or F3).

Table S1. Primers used for quantitative real time PCR analysis

Gene	Primer	Sequence
**Mouse GAPDH**	Forward	GACTTCAACAGCAACTCCCAC
	Reverse	TCCACCACCCTGTTGCTGTA
**Mouse Dnmt1**	Forward	GTCGGACAGTGACACCCTTT
	Reverse	TTTAGTGGGGCCCTTCGTG
**Mouse Dnmt3b**	Forward	CCTCCGACGCAGGAA AGATT
	Reverse	ACTTGGGTGGCTCCTGAAAT
**Mouse IL-4**	Forward	GCATGGAGTTTTCCCATGTT
	Reverse	AGATGGATGTGCCAAACGTC
**Mouse IL-5**	Forward	CTCTGTTGACAAGCAATGAGACG
	Reverse	TCTTCAGTATGTCTAGCCCCTG
**Mouse IL-13**	Forward	CCTCTGACCCTTAAGGAGCTTAT
	Reverse	CGTTGCACAGGGGAGTCT
**Mouse IL-33**	Forward	ATTTCCCCGGCAAAGTTCAG
	Reverse	AACGGAGTCTCATGCAGTAGA
**Mouse IL-10**	Forward	CAGAGCCACATGCTCCTAGA
	Reverse	GTCCAGCTGGTCCTTTGTTT
**Mouse Ccl11/Eotaxin**	Forward	TTCTATTCCTGCTGCTCACGG
	Reverse	AGGGTGCATCTGTTGTTGGTG
**Mouse Ccl3/MIP-1α**	Forward	ACTGCCTGCTGCTTCTCCTACA
	Reverse	AGGAAAATGACACCTGGCTGG
**Mouse CXCL1/KC**	Forward	CTGGGATTCACCTCAAGAACATC
	Reverse	CAGGGTCAAGGCAAGCCTC
**Mouse CXCL2**	Forward	TGTCCCTCAACGGAAGAACC
	Reverse	CTCAGACAGCGAGGCACATC
**Mouse Muc5ac**	Forward	CTGTGACATTATCCCATAAGCCC
	Reverse	AAGGGGTATAGCTGGCCTGA
**Mouse Muc5b**	Forward	CATGGATGGCTGCTTCTGT
	Reverse	TAAATTCGGCCACCGTGT
**Mouse GATA3**	Forward	CCCATTACCACCTATCCGCC
	Reverse	GTTCACACACTCCCTGCCTT
**Mouse MyD88**	Forward	CCACTCGCAGTTTGTTGGAT
	Reverse	TCCTCACGGTCTAACAAGGC

Table S2 The parameters involved in inflammatory responses with statistical difference between three size-fractionated ultrafine and fine atmospheric particulate matter groups in OVA-induced asthma model

Relevant parameters involved in asthma inflammation [a]	OVA/PM groups that have significant difference between them [a]
HO-1 expression in RAW264.7 cells (5 h)	OVA/F1 vs OVA/F2, OVA/F1 vs OVA/F3
HO-1 expression in RAW264.7 cells (18 h)	OVA/F1 vs OVA/F2, OVA/F3 vs OVA/F2
HO-1 expression in lung tissues	OVA/F2 vs OVA/F1, OVA/F2 vs OVA/F1
Dnmt1 expression in RAW264.7 cells	OVA/F1 vs OVA/F2, OVA/F1 vs OVA/F3
Dnmt3b expression in RAW264.7 cells	OVA/F1 vs OVA/F2, OVA/F1 vs OVA/F3
Dnmt3b expression in lung tissues	OVA/F3 vs OVA/F1, OVA/F3 vs OVA/F1
Lymphocyte in BALF	OVA/F1 vs OVA/F2, OVA/F1 vs OVA/F3
Neutrophil in BALF	OVA/F1 vs OVA/F2, OVA/F1 vs OVA/F3
Eosinophil in BALF	OVA/F1 vs OVA/F3, OVA/F2 vs OVA/F3
Total IgE of serum	OVA/F2 vs OVA/F1, OVA/F2 vs OVA/F1
Muc5ac expression in lung tissues	OVA/F2 vs OVA/F1, OVA/F2 vs OVA/F1
Muc5b expression in lung tissues	OVA/F1 vs OVA/F3, OVA/F2 vs OVA/F3
PAS staining	OVA/F1 vs OVA/F3, OVA/F2 vs OVA/F3
IL-4 expression in lung tissues	OVA/F1 vs OVA/F2, OVA/F1 vs OVA/F3
IL-5 expression in lung tissues	OVA/F1 vs OVA/F3, OVA/F2 vs OVA/F3
IL-13 expression in lung tissues	OVA/F1 vs OVA/F2, OVA/F1 vs OVA/F3
IL-33 expression in lung tissues	OVA/F1 vs OVA/F2, OVA/F1 vs OVA/F3
IL-10 expression in lung tissues	OVA/F1 vs OVA/F2, OVA/F1 vs OVA/F3
CXCL1/KC expression in lung tissues	OVA/F1 vs OVA/F3, OVA/F2 vs OVA/F3

[a] Refers to statistical difference analysis of relevant parameters involved in inflammatory responses between OVA and PM groups. The OVA/PM group(s) that have significant difference between them ($p<0.05$) is (are) listed in the table.
